# Particulate multivalent presentation of the receptor binding domain induces protective immune responses against MERS-CoV

**DOI:** 10.1080/22221751.2020.1760735

**Published:** 2020-05-29

**Authors:** Nisreen M. A. Okba, Ivy Widjaja, Brenda van Dieren, Andrea Aebischer, Geert van Amerongen, Leon de Waal, Koert J. Stittelaar, Debby Schipper, Byron Martina, Judith M. A. van den Brand, Martin Beer, Berend-Jan Bosch, Bart L. Haagmans

**Affiliations:** a Department of Viroscience, Erasmus Medical Center, Rotterdam, The Netherlands; b Virology Division, Department of Infectious Diseases and Immunology, Faculty of Veterinary Medicine, Utrecht University, Utrecht, The Netherlands; c Institute of Diagnostic Virology, Friedrich-Loeffler-Institut, Insel Riems, Germany; d Viroclinics Biosciences BV, Rotterdam, The Netherlands

**Keywords:** Vaccine, MERS-coronavirus, spike, i301, lumazine synthase, spytag-spycatcher, rabbit, SARS-CoV-2

## Abstract

Middle East respiratory syndrome coronavirus (MERS-CoV) is a WHO priority pathogen for which vaccines are urgently needed. Using an immune-focusing approach, we created self-assembling particles multivalently displaying critical regions of the MERS-CoV spike protein ─fusion peptide, heptad repeat 2, and receptor binding domain (RBD) ─ and tested their immunogenicity and protective capacity in rabbits. Using a “plug-and-display” SpyTag/SpyCatcher system, we coupled RBD to lumazine synthase (LS) particles producing multimeric RBD-presenting particles (RBD-LS). RBD-LS vaccination induced antibody responses of high magnitude and quality (avidity, MERS-CoV neutralizing capacity, and mucosal immunity) with cross-clade neutralization. The antibody responses were associated with blocking viral replication and upper and lower respiratory tract protection against MERS-CoV infection in rabbits. This arrayed multivalent presentation of the viral RBD using the antigen-SpyTag/LS-SpyCatcher is a promising MERS-CoV vaccine candidate and this platform may be applied for the rapid development of vaccines against other emerging viruses such as SARS-CoV-2.

## Introduction

Emerging zoonotic viruses, such as severe acute respiratory syndrome coronavirus (SARS-CoV) and Middle East respiratory syndrome coronavirus (MERS-CoV) have been able to cross the species barrier posing a threat to the human population. MERS-CoV causes severe respiratory disease and fatalities in humans [1,2], and the virus is continuously introduced into the human population through infected dromedary camels, the viral reservoir with resulting outbreaks [3]. The wide geographical distribution of this viral reservoir, the high case-fatality rate in humans (35%), and the lack of treatment and licensed vaccines, make the virus a threat to the human population. This has put MERS-CoV on the recent WHO list of diseases having an epidemic or even pandemic potential for which countermeasures are lacking and are urgently needed [4].

Vaccination is potentially one of the most effective ways to prevent the ongoing MERS-CoV outbreaks. Several MERS-CoV vaccine candidates have been developed using different platforms including inactivated, live-attenuated, and subunit vaccines [5]. Compared to other vaccine production platforms, recombinant subunit proteins have a higher safety profile, are relatively faster and easier to produce, and can be scaled-up in a more cost-effective manner; nonetheless, they tend to induce lower levels of protective immunity [6]. The use of self-assembling multimeric protein scaffold particles (MPSP) to present antigens in a multivalent virus-mimicking manner (size, repetitiveness, and geometry), has been shown to enhance vaccine-induced immune responses [7–11], and to offer advantages over other multimeric antigen presentation platforms (reviewed in [12]). Both lumazine synthase (LS) and I3-01 (I3) can self-assemble into 60-meric particles, which can be expressed in *E. coli* and have been used as scaffolds for development of multimeric vaccines with improved immune responses compared to monomeric forms [13–15]. An LS-based HIV vaccine, (eOD-GT8), has recently advanced to a phase I human clinical trial (NCT03547245). Linking of antigens to these MPSP can be achieved through several mechanisms; as e.g. genetic fusion or the SypTag-SpyCatcher (ST/SC) system [16]. While the former requires the antigen and scaffold to be produced in the same expression system, the latter allows each to be expressed in its suitable system harnessing a rapid post-translational “plug-and-play” assembly. This is advantageous, allowing scaffold-SC to be produced at scalable levels in *E. coli* and SpyTagged glycosylated antigens such as viral surface proteins to be produced in its optimal system, such as mammalian or insect cells. The antigen-ST can then be multivalently displayed on the surface of the SC-scaffolds through the spontaneous formation of a stable isopeptide bond. This can be a platform for rapid vaccine manufacturing in case of epidemics or pandemics, to create optimized vaccines at reduced costs and also with reduced development times.

The MERS-CoV spike (S) protein is the main target for subunit vaccine development [5] It assembles as a homotrimer and consists of an N-terminal head (S1 subunit) and a C-terminal stalk (S2 subunit). The S1 subunit mediates virus attachment and entry through its N-terminal S1^A^ domain and its C-terminal receptor binding domain (RBD), respectively [17,18]. The S1^A^ domain binds sialic acids, a viral attachment factor, while the RBD binds to the viral receptor, dipeptidyl peptidase 4 (DPP4). Following attachment and entry, the S2 subunit mediates viral fusion to the host cell through its fusion machinery; comprised of the fusion peptide (FP) and the two heptad repeats – HR1 and HR2 [19]. MERS-CoV neutralizing antibodies (Abs) mainly recognize epitopes in the RBD of the spike head S1 subunit; and to a lower extent, epitopes in the sialic acid binding domain and the fusion-mediating more conserved S stalk (S2). Nonetheless, antibodies directed against the sialic acid binding S1^A^ domain or the more conserved S2 subunit, although subdominant, may protect against MERS-CoV [20,21].

Immune focusing can enhance immune responses to subdominant regions [22]. In the current study, using LS and I3 self-assembling particles, we evaluated whether immune focusing and multivalent presentation can induce immune responses to the more sequence-conserved S2 regions: FP and HR2. Furthermore, using a SypTag/SpyCatcher system and LS particles, we tested whether immune focusing with/without multivalent presentation of the viral RBD can lead to enhanced protection against a MERS-CoV challenge in rabbits.

## Materials and methods

### Protein design and expression

Expression constructs were cloned using standard PCR methods. The gene encoding the 6,7-dimethyl-8-ribityllumazine synthase (LS; GenBank accession no. WP_010880027.1) of *A. aeolicus* was synthesized using human-preferred codons obtained from GenScript USA, Inc, as described previously [17]. The cysteine at position 37 and asparagine at position 102 of LS were mutated to alanine and glutamine, respectively. The gene encoding I3-01 (I3; PDB 5KP9, amino acid residues 19–222) derived from *Thermotoga maritima* was synthesized using human-preferred codons obtained from GenScript USA. The gene fragments encoding the ΔN1SpyCatcher (SC; UniProt accession no. AFD50637.1; amino acid residues 48–139; [23]) and SpyTag (ST; UniProt accession no. WP_129284416.1; amino acid residues 981–994) based on the Cna B-type domain-containing protein of *Streptococcus pyogenes* were synthesized using human-preferred codons obtained from GenScript USA, Inc. The LS and I3 gene constructs were cloned into the pGEX-2 T bacterial expression vector (Sigma Aldrich).

To generate the HR2-LS expression vector, the HR2 region (amino acid residues 1215–1287) encoding sequence of the MERS-CoV S gene (accession no. NC_019843) was ligated in-frame with an N-terminal sequence encoding a CD5 signal sequence and streptag tag purification tag, and with a C-terminal sequence encoding the LS via a linker, and subsequent cloned into the pCAGGS mammalian expression vector.

To generate the I3-HR2 expression vector, the heptad repeat 2 encoding region (HR2, amino acid residues 1215–1287) of the MERS-CoV S gene was ligated in-frame with an N-terminal sequence encoding the I3-01 and a C-terminal streptag purification tag interspaced with a linker, and subsequent cloned into the pGEX-2 T bacterial expression vector (Sigma Aldrich).

To generate the FP-I3 and FP-LS expression vectors, the fusion peptide (FP; amino acid residues 884–898) encoding sequence of the MERS-CoV S gene was ligated in-frame with an N-terminal sequence encoding the I3-01 or LS, and a C-terminal Streptag purification tag and subsequently cloned into the pGEX-2 T bacterial expression vector (Sigma Aldrich).

To generate the RBD-ST expression vector, the MERS-RBD (amino acid residues 358–588) encoding sequence of the MERS-CoV S gene was ligated in-frame with an N-terminal sequence encoding a CD5 signal sequence and with a C-terminal sequence encoding the ST followed by a double Streptag, and subsequently cloned into the pCAGGS mammalian expression vector.

To generate the LS-SC expression vector, the codon optimized SC sequence equipped with an N-terminal FLAG-tag (DYKDDDDK) was cloned to the N-terminus of the LS sequence in the pET15b bacterial expression vector (Novagen).

All protein sequences are provided in Supplementary Figures S1 and S2.

### Mammalian expression

Mammalian expression of the HR2-LS and RBD-ST constructs was done, as described previously [17]. In short, expression plasmids were polyethylenimine (PEI)-transfected into 60% confluent HEK-293 T cells for 6 h, after which transfections were removed and medium was replaced with 293 SFM II-based expression medium (Gibco Life Technologies) and incubated at 37 °C in 5% CO_2_. Tissue culture supernatants were harvested 5–6 d post transfection, and expressed proteins were purified using StrepTactin Sepharose beads (IBA) according to the manufacturer’s instruction.

### Bacterial protein expression

BL21 cells (Novagen) were transformed with pGEX-2 T expression vectors and grown in 2× yeast-tryptone medium to log phase (OD_600_ ∼1.0) and subsequently induced by adding IPTG (isopropyl-β-d-thiogalactopyranoside) (GIBCO BRL) to a final concentration of 1 mM. Two hours later, the cells were pelleted, resuspended in 1/25 volume of 10 mM Tris (pH 8.0)-10 mM EDTA-1 mM phenylmethylsulfonyl fluoride, and sonicated on ice (five times, 2 min each). The cell homogenates were centrifuged at 20,000 × g for 60 min at 4°C. Proteins were purified from the cell lysate supernatant using StrepTactin Sepharose beads (IBA) according to the manufacturer’s instruction.

All purified proteins were analyzed on a 12% SDS/PAGE gel under reducing conditions and stained with GelCodeBlue stain reagent (Thermo Scientific). Purified proteins were stored at 4°C until further use.

Expression of the FLAG-LS-SC was performed as described above with the following modifications: 1) Cells were treated with 1 mg/ml lysozyme in lysis buffer (50 mM Tris-HCl, 150 mM NaCl, 1% Triton X-100) for 1 h at room temperature prior to sonification on ice. 2) Purification was performed using ANTI-FLAG® M2 Affinity Gel (Sigma Aldrich) as recommended by the manufacturer. Purified proteins were dialyzed against 1x TBS buffer (50 mM Tris-HCl, 150 mM NaCl, pH 7.4) and stored at −80°C until further use.

### Rabbit immunizations

Rabbit immunizations and challenge were carried out at Viroclinics Bioscience B.V. under permit no. AVD277002015283-WP03, using BSL-3 containment facilities. Female New Zealand White rabbits (Envigo, Venray, the Netherlands) of 11 weeks age were assigned to six groups (i-vi) of five animals each. Immunizations were performed intramuscularly with either i) HR2-LS, ii) FP-LS, iii) LS, at day 0 and boosted with either i) HR2-I3, ii) FP-I3, iii) I3 on day28 or iv) PBS, v) RBD + LS, vi) RBD-LS on days 0 and 28. Each animal received each time 15 µg of antigen adjuvanted with Adjuplex (5%; Sigma-Aldrich, Zwijndrecht, the Netherlands) in a total volume of 500 µL. Three weeks after the last vaccination (day 49 of the study), all animals were challenged intranasally under anesthesia with MERS-CoV (10^6^ 50% tissue culture infectious dose (TCID_50_) MERS-CoV EMC strain (accession no. NC_019843) in a volume of 1 mL divided over both nostrils). The animals were euthanized on day 4 post-challenge (day 53 of the study). Serum samples were collected on days 0, 28, and 46. Nasal swabs were collected on day 46 (pre-challenge) and on days 1 through 4 post-challenge. Following euthanasia, lungs were examined for gross pathology and lung tissue samples were collected for virus detection, and in 10% formalin histopathology and immunohistochemistry.

### Enzyme-linked immunosorbent assay (ELISA)

Antigen-binding and anti-LS (scaffold) antibodies produced after vaccination were tested in the sera collected at different time points as well as in pre-challenge nasal swabs using ELISA. Costar high-binding 96-well ELISA plates were coated overnight at 4°C with 1 µg/ml of either recombinant LS, MERS-CoV S1 or S2 proteins in PBS. The plates were washed with PBS and blocked for 1 hr using 1%BSA/0.5%Tween-20/PBS. Following blocking, diluted samples (1:100 or serially diluted) were added and further incubated for 1 hr. The plates were then washed and and probed with an HRP-labeled goat anti-rabbit Ig (1:2000, Dako) secondary antibody. TMB was used for signal development and the absorbance of each sample was measured at 450 nm (OD_450_).

### Antibody avidity ELISA

Antibody avidity was assessed using an ammonium thiocyanate (NH_4_SCN)-displacement ELISA. This was carried out as described above using serum dilutions containing same level of S1 absorbance units added in triplicates. Following serum incubation and washing, NH_4_SCN (0–5 M) was added to the wells for 15 min. The plates were then washed and further developed as described above. The concentration of NH_4_SCN resulting in a 50% reduction in signal was taken as the avidity index (IC_50_).

### ELISA analysis of immunogen binding by antibodies

To confirm the antigenicity of the RBD-LS particles, we tested its binding to well-characterized monoclonal antibodies binding conformational RBD epitopes [20]. Human monoclonal antibodies 7.7G6, 1.6F9, 1.2G5, 1.8E5, 4.6E10 targeting the receptor binding domain of the MERS-CoV spike protein were produced and purified as described earlier [20]. NUNC Maxisorp plates (Thermo Scientific) were coated with the RBD-LS antigen at 100 ng /well at 4°C overnight. Plates were washed three times with PBS containing 0.05% Tween-20 and blocked with PBS with 5% Protifar in PBS containing 0.1% Tween-20 at room temperature for 2 h. Four-folds serial dilutions of mAbs starting at 10 µg/ml (diluted in blocking buffer) were added and plates were incubated for 1 h at room temperature. Plates were washed three times and incubated with HRP-conjugated goat anti-human secondary antibody (ITK Southern Biotech) diluted 1:2000 in blocking buffer for one hour at room temperature. HRP activity was measured at 450 nm using tetramethylbenzidine substrate (BioFX) and an ELISA plate reader (EL-808, Biotek).

### Plaque reduction neutralization assay

The presence of MERS-CoV neutralizing antibodies in the sera and nasal swabs of vaccinated animals was tested using a plaque reduction neutralization assay (PRNT). Heat –inactivated two-fold serially diluted samples (starting 1:10) were mixed 1:1 with 400 PFU of MERS-CoV (EMC/2012) and incubated for one hour. The mix was then overlaid on HuH-7 cells in 96-well plates. Following one hour of incubation, the mix was removed and the cells were incubated for 8 hr. The cells were then fixed, permeabilized and stained using a mouse anti-MERS-CoV N protein monoclonal antibody (Sino Biological) followed by an HRP-labelled goat anti-mouse IgG1 (SouthernBiotech). The signal was developed using a precipitate forming peroxidase substrate (True Blue, KPL). The ImmunoSpot® Image analyzer (CTL Europe GmbH) was used to count the number of infected cells per well. The neutralization titre of each serum sample was determined as the reciprocal of the highest dilution resulting in a ≥50% (PRNT_50_) or ≥90% (PRNT_90_) reduction in the number of infected cells. A titre of ≥ 20 was considered to be positive.

### Viral RNA detection

To evaluate the protective efficacy of vaccination against MERS-CoV challenge, nasal swabs, and homogenated lung tissues were tested for the presence of MERS-CoV RNA using RT-qPCR for and for the presence of infectious virus by virus titration.

The presence of viral RNA in nasal swabs and lung tissues was tested using UpE RT-qPCR as previously described [24]. RNA was extracted from samples using Magnapure LC total nucleic acid isolation kit (Roche). RNA amplification and quantification were carried out using a 7500 Real-Time PCR System (Applied biosystems). Samples with a C_t_ value <40 were considered positive. RNA dilutions extracted from a MERS-CoV stock of known titre was used to generate a standard curve in order to calculate the TCID_50_ equivalent of RNA detected in samples. Concentrations of viral RNA in lung tissue are expressed in as TCID_50_ equivalents per gram tissue (TCID_50_ eq/g), and in the nasal swabs as TCID_50_ eq/mL.

### Virus titration

The presence of MERS-CoV infectious viral particles in respiratory tract samples (nasal swabs and lung tissue homogenates) was detected by titration on Vero cells as described previously [24]. Briefly, 10-fold serially diluted samples (starting undiluted) were overlaid on Vero cells and the plates were incubated for five days at 37°C and the cytopathic effect was recorded. Infectious virus titres in lung tissue are expressed as TCID_50_ per gram tissue (TCID_50_/g), and infectious virus titre in nose swabs are expressed as TCID_50_/mL.

### Histopathology and immunohistochemistry

Lung tissue samples were collected in formalin and embedded in paraffin for pathological analysis. Hematoxylin-eosin staining was carried out for histopathological analysis. The presence of MERS-CoV nucleoprotein was detected by immunohistochemistry as previously published [24].

### Statistical analysis

Statistical analyses were performed using Prism 7 (GraphPad Software Inc, USA). Data were compared using Mann-Whitney U test or Student’s t-test. *P*-values < 0.05 were considered significant.

## Results

### Generation of MERS-CoV spike particles

**Figure 1.**
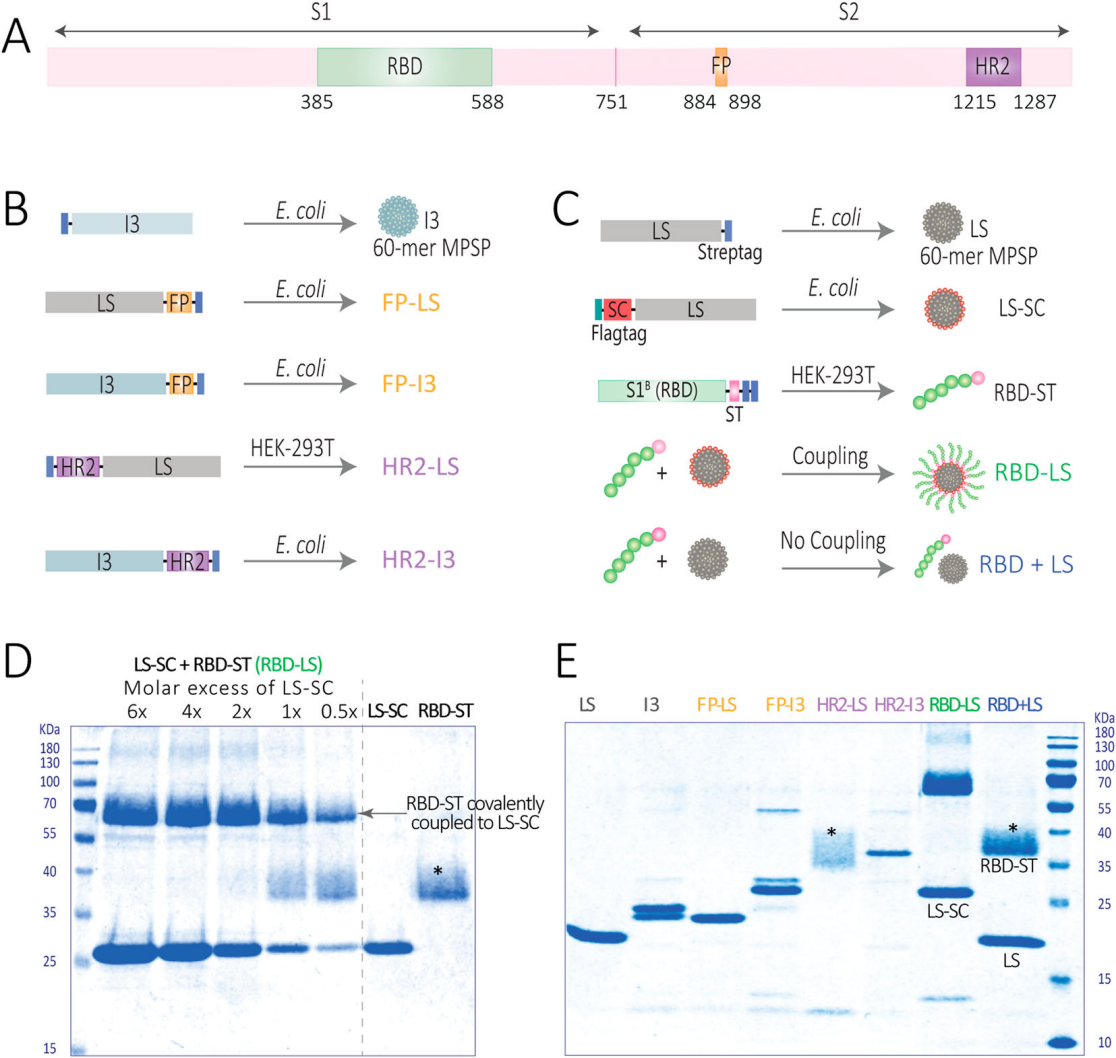
Generation of multimeric protein scaffold particles (MPSP)- based vaccines used in this study. (A) Schematic diagram of the MERS-CoV spike (S) protein mapping regions selected for vaccine generation; the receptor binding domain (RBD), the fusion peptide (FP) and heptad repeat 2 (HR2). (B, C) Schematic diagram illustrating the construct design and production of the lumazine synthase (LS) and I3-01 (I3)-based self-assembling MPSP vaccines. (D) Reducing SDS-PAGE showing generation of RBD-LS by covalent coupling of RBD-SpyTag (RBD-ST) and LS-SpyCatcher (LS-SC) at different molar ratios of LS-SC: RBD-ST with the last two lanes showing each in its free (uncoupled) form. (E) Reducing SDS-PAGE analysis of immunogens used in this study. The size of each protein (KDa) is given in Supplementary Table S1. * Fuzzy bands due to heterogeneous glycosylation of HR2 or RBD.

Particulate multivalent antigen display can enhance immunogenicity through different mechanisms, allowing for induction of immune responses against otherwise weakly immunogenic antigens [7,25]. We sought to design antigens capable of inducing strong immune responses against critical parts of the viral entry and fusion machinery within the MERS-CoV spike protein through immune focusing and multivalent presentation on self-assembling particles (Figure 1). Within the S1 subunit, the RBD is the main target for the induction of neutralizing antibodies and has been used to develop several vaccine candidates for MERS-CoV [5,26]. Indeed, the immunogenicity of RBD can be enhanced by its presentation on ferritin nanoparticles [27]. Likewise, the fusion peptide (FP) and the HR2, which show a high degree of sequence conservation among CoVs relative to the RBD, play crucial roles in the CoV spike-mediated fusion machinery, and can be targets for CoV protective antibodies [28–32]. Genetic fusion was chosen for FP and HR2, due to their small size, whereas the ST/SC system was used for RBD display on particles to ensure correct folding of the protein.

Two 60-meric hyperstable self-assembling particles with icosahedral symmetry were used for multivalent display of MERS-CoV domains. The lumazine synthase (LS) particle, an icosahedron with a diameter of 15 nm (PMID: 23539181) and the I3-01 (I3) particle, a dodecahedron with a diameter of 25 nm (PMID: 27309817). The N- and C-termini of both scaffolds are surface exposed, providing a platform to multivalently present (antigenic) domains. Two functional segments of the S2 subunit of the MERS-CoV spike protein were genetically fused to these nanoparticles; the fusion peptide containing region (amino acid residues 884–898) and the HR2 containing region (amino acid residues 1215–1287) (Figure 1B, Supplementary Figure S1). Chimeric nanoparticles were purified after expression in eukaryotic (mammalian) or prokaryotic systems (Figure 1).

### Generation of multimeric RBD-ST/LS-SC (RBD-LS)

In addition, we used the SpyTag/SpyCatcher system to multivalently display the MERS-CoV RBD on LS nanoparticle via covalent bonding [8]. For this purpose, the SpyCatcher (SC) was genetically fused to LS and expressed and purified from *E. coli*. The SpyTag (ST) was genetically fused to the MERS-RBD (amino acid residues 358–588) and expressed and purified from HEK-293 T cells (Figure 1C). RBD-ST was incubated with LS-SC in different molar ratios to assess the optimal coupling of both components. A 1:2 molar ratio of RBD-ST and LS-SC allowed the optimal coupling of all of the provided RBD-ST antigens to the SC-LS particles (Figure 1D). The resulting conjugation products were used for immunization. In order to assess the effect of the particle-based multivalent antigen display on immunogenicity, a mixture of non-coupled RBD-ST and LS (without SC) was taken along for immunization in the same molar ratio. All particulate preparations displaying MERS-S antigenic domains (genetically fused or SC/ST coupled) were analyzed by SDS-PAGE (Figure 1E, Supplementary Table S1), confirming their molecular integrity. We further confirmed the antigenicity of the RBD-LS particles by testing their capacity to bind monoclonal antibodies directed against conformational epitopes on the RBD [20] using ELISA. All antibodies bound to RBD-LS in a dose dependant manner (Figure S3) indicating that the RBD is correctly folded confirming its antigenicity.

### Immunogenicity of particulate MERS-CoV spike vaccines in rabbits

**Figure 2.**
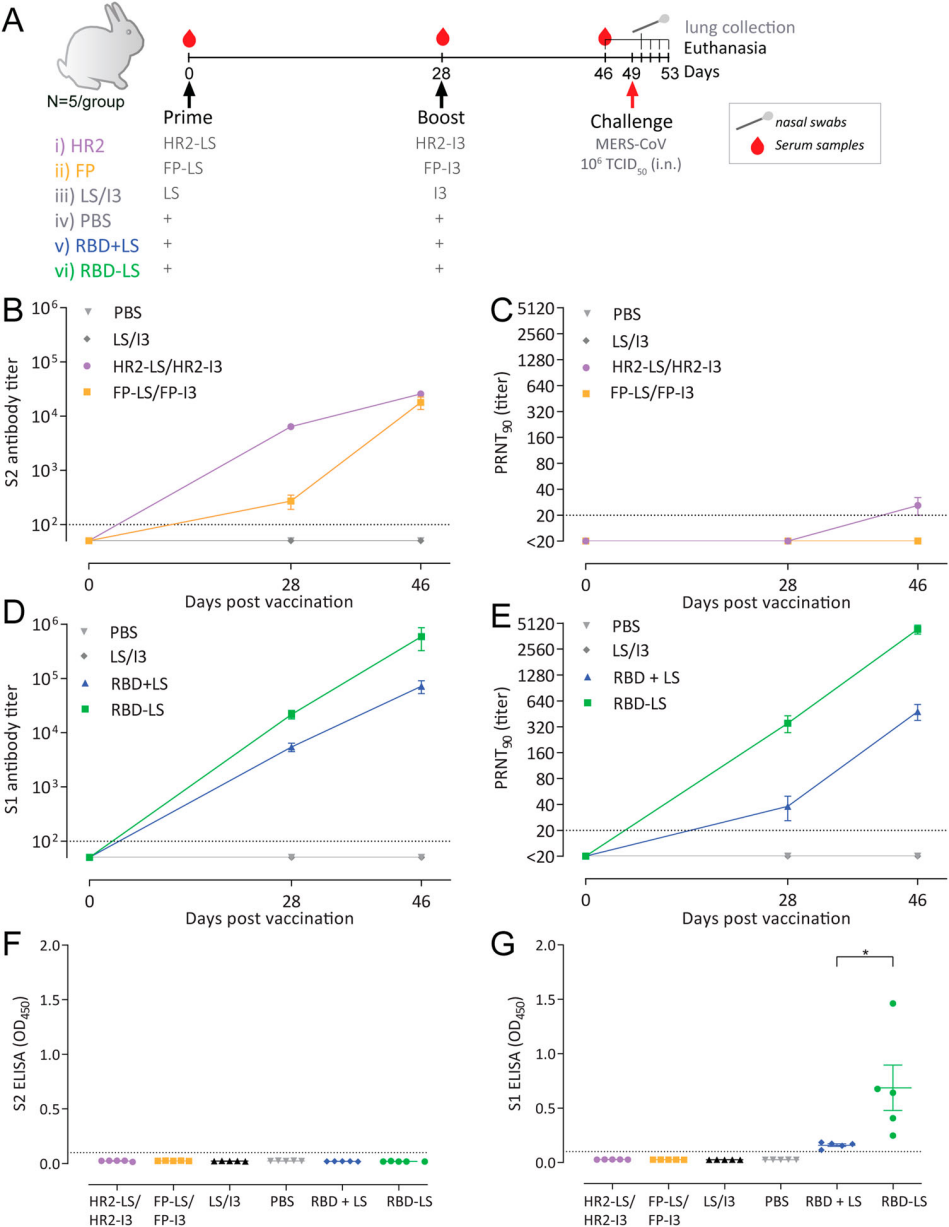
Immunogenicity of MERS-CoV spike MPSP vaccines. (A) Vaccination scheme for rabbit immunizations. Six groups of rabbits (5/group) were vaccinated in a prime/boost regimen with 15 μg of adjuvanted vaccine at 4-week interval and challenged with MERS-CoV (EMC strain; accession no. NC_019843) 3 weeks post-boost. Anti-MERS-CoV spike S2 (B) and S1 (D) IgG titres measured by ELISA in rabbits at different time points. Shown is the mean ± s.e.m. antibody titres from five rabbits per group. (C,E) MERS-CoV neutralizing antibody titres measured by a 90% reduction in a plaque reduction neutralization assay (PRNT_90_). (B-E) Shown is the mean ± s.e.m. of five rabbits per group. (F,G) Vaccine-induced antibodies in nasal swabs of vaccinated rabbits. Anti MERS-CoV S2 (F) and S1 (G) antibody responses in the nasal swabs (tested at a 1:50 dilution) of vaccinated rabbits pre-challenge (three weeks post-boost). The difference in antibody responses between monomeric (RBD + LS) and multimeric (RBD-LS) RBD was tested for statistical significance using a student’s t-test, with asterisks indicating the level of significance. *P ≤ 0.05. Error bars indicate mean ± s.e.m. The dotted lines represent the limits of detection. HR2, heptad repeat 2; FP, fusion peptide; LS, lumazine synthase 60-meric particles; I3, I3-01 60-meric particles; RBD, receptor binding domain; RBD + LS, monomeric uncoupled RBD; RBD-LS, multimeric RBD coupled to LS through covalent SpyTag/SpyCatcher.

We then evaluated the immunogenicity of the multimeric spike antigens using six groups of rabbits (*n* = 5 per group), which were intramuscularly immunized twice at a 4-week interval (Figure 2A). The LS/I3 and PBS immunized groups served as controls.

After the first immunization, we detected antibody responses against the corresponding S subunit (S1 or S2) in the vaccinated rabbits. while the control groups remained negative (Figure 2B–E). Endpoint antibody titres for the vaccinated groups are shown as geometric mean titres (GMT) in Supplementary Table S2. The antibody responses were further boosted after the second immunization in all groups, while no responses were detected in the control groups, confirming the immunogenicity of the tested antigens in rabbits.

Anti-S2 antibody responses were detected in the HR2 and FP vaccinated groups with weak to no MERS-CoV neutralizing capacity (Figure 2B,C). Only HR2 vaccination induced low levels of MERS-CoV neutralizing antibodies (PRNT_90_ titres: 20 – 40) in 4/5 rabbits; all 5 had MERS-CoV neutralizing antibodies at a 50% cut-off (data not shown).

Likewise, both the monomeric RBD (RBD + LS) and the multimeric RBD-LS were immunogenic and elicited high S1-specific antibody titres which were further boosted after the second immunization. The RBD-LS-induced S1 antibody titres were significantly higher than those induced by the monomeric RBD following the prime- as well as booster-vaccination (*P* = 0.0397 and *P* = 0.0317, respectively by Mann-Whitney U test) (Figure 2D). Multimeric RBD-LS vaccination elicited higher MERS-CoV neutralizing antibodies, a main correlate of protection, than the monomeric RBD + LS when tested for live virus neutralization using PRNT_90_ assay (*P* = 0.0109, and *P* = 0.0079, post-prime and boost, respectively by Mann-Whitney U test) (Figure 2E). The vaccine induced antibodies were able to neutralize clade A (EMC/2012 strain; Figure 2E) as well as the more recently circulating clade B (Qatar15/2015 strain; Supplementary Figure S4) viruses. The spike protein of the former strain differs from the clade A EMC/2012 strain in two positions; T95S and Q1020R.

Following a single immunization, binding antibody titres were four-fold higher and neutralizing antibodies were eleven-fold higher in the coupled multimeric RBD-LS group than in the uncoupled monomeric RBD + LS (Supplementary Table S2). Three weeks after the boost, binding antibody responses were seven-fold higher (*P* = 0.0079, Mann-Whitney U test) and neutralizing antibodies were ten-fold higher (*p* = 0.0079, Mann-Whitney U test) in the coupled RBD-LS group than in the uncoupled RBD + LS (Figure 2D, E Supplementary Table S2). Additionally, we tested for vaccine induced mucosal immunity in the respiratory tract of vaccinated rabbits pre-challenge (Day 49) using ELISA. MERS-CoV specific antibodies were only detected in the nasal swabs of the groups vaccinated with conjugated or non-conjugated RBD (Figure 2 F,G). Antibody responses detected in the RBD-LS vaccinated group were higher than those in the RBD + LS vaccinated group (*P* = 0.0357, Student’s t-test). This demonstrates that RBD-LS induces improved local mucosal immune responses compared to the monomeric RBD. Thus, vaccination with the newly produced RBD-LS MERS-CoV MPSP vaccines induce a robust immune response.

### Avidity of RBD-LS induced antibodies

**Figure 3.**
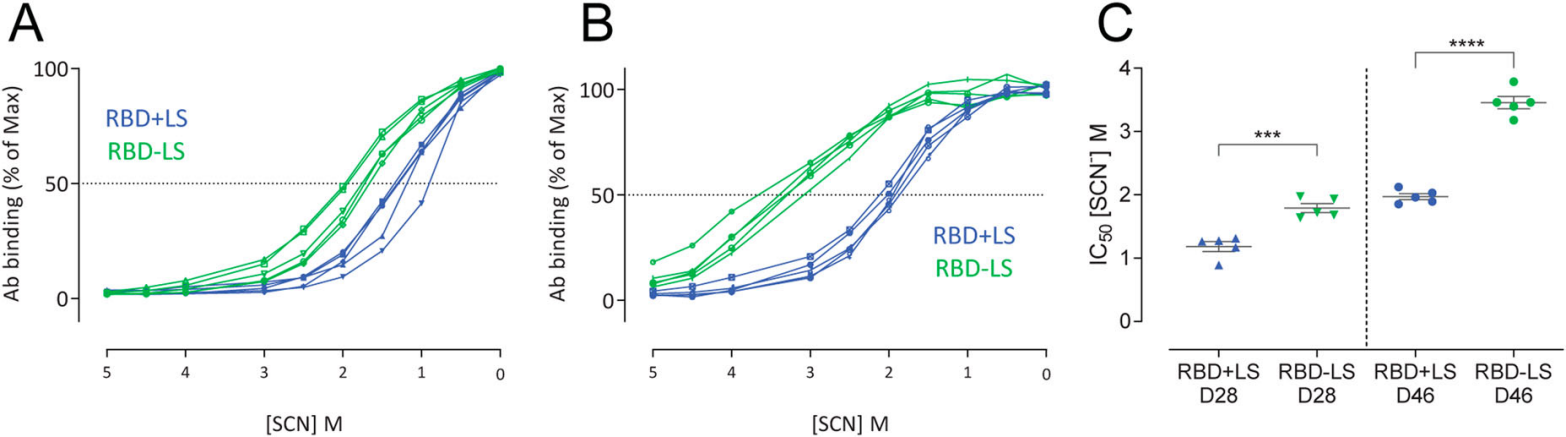
Avidity of vaccine-induced serum antibody responses. The avidity of serum IgG antibody responses after one (A, Day 28) and two immunizations (B, Day 46) with either monomeric RBD (RBD + LS, blue, *n* = 5) or multimeric RBD (RBD-LS, green, *n* = 5) was assessed using ammonium thiocyanate (SCN) avidity ELISA. (A, B) The percentage of serum antibodies bound following the addition of different concentration of SCN was used to determine (C) the avidity index (IC_50_). The difference in serum avidity between both groups was tested for statistical significance using a student’s t-test, with asterisks indicating the level of significance. ***P ≤ 0.001, ****P ≤ 0.0001. Error bars indicate mean ± s.e.m.

The avidity of MERS-CoV spike-specific antibodies in the monomeric versus the multimeric RBD vaccinated groups was analyzed at days 28 (4 weeks after prime) and 46 (3 weeks after boost) using an ammonium thiocyanate (NH_4_SCN)-displacement ELISA [33]. The avidity index IC_50_ was determined for each vaccinated rabbit and compared between the two groups. The avidity of the S1-specific antibody responses was higher following RBD-LS vaccination compared to the monomeric RBD + LS vaccination (*p *< 0.0001, Student’s t-test) (Figure 3), indicating that a multimeric RBD-LS vaccine can induce antibody responses of both higher quantity and quality (Figures 2D,E and 3).

### Antibody responses to lumazine synthase scaffolds

**Figure 4.**
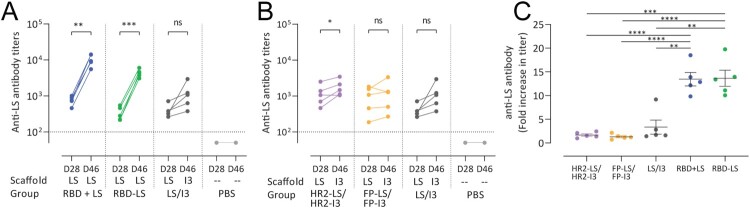
Anti-scaffold antibody responses in sera of vaccinated rabbits. Anti-lumazine synthase (LS) scaffold antibody titres following (A) homologous prime boost in monomeric RBD + LS and multimeric RBD-LS vs heterologous LS/I3 prime boost in control LS/I3 as well as (B) HR2-LS/I3 and FP-LS/I3. Shown are (average ± s.e.m. of *n* = 5 rabbit/group) antibody titres 4 weeks after prime (day 28, D28) and 3 weeks after boost (day 46, D46) as measured by ELISA. (C) Fold increase (from prime, day 28) in anti-LS antibody titres following boost vaccination (day 46). A paired t-test was performed to determine significant increases in antibody titres post-prime and post-boost within groups (A, B), and an unpaired t-test was performed to determine significant changes in titres between groups (C), with asterisks indicating the level of significance.*P ≤ 0.05, **P ≤ 0.01, ***P ≤ 0.001, ****P ≤ 0.0001. The dotted lines represent the limits of detection. HR2, heptad repeat 2; FP, fusion peptide; LS, lumazine synthase 60-meric particles; I3, I3-01 60-meric particles; RBD, receptor binding domain; RBD + LS, monomeric uncoupled RBD; RBD-LS, multimeric RBD coupled to LS through covalent SpyTag/SpyCatcher.

In addition to evaluating anti-S (antigen) responses, we also tested for the induction on LS-specific (scaffold) antibodies. Antibody responses were elicited against the LS-particle in all groups except the PBS group, indicating that the particle was accessible and not sterically hidden by antigens displayed on its surface; even when RBD was displayed on its surface using SpyTag:SpyCatcher linkage (Figure 4). Despite that, antigen-specific responses were not adversely affected by the presence of these anti-scaffold antibodies, as demonstrated by the booster effect after the second immunization (Figure 2D,E). Nonetheless, we tested whether a heterologous scaffold boost could help in minimizing such anti-scaffold responses using an LS/I3 prime-boost scheme. Using this approach, we found no significant increase in anti-scaffold antibody responses compared to the homologous prime-boost scheme (Figure 4C). This indicates that a heterologous scaffold prime-boost approach could be advantageous for limiting unnecessary anti-scaffold responses.

### Efficacy of RBD-LS in preventing virus shedding and infection in rabbits

**Figure 5.**
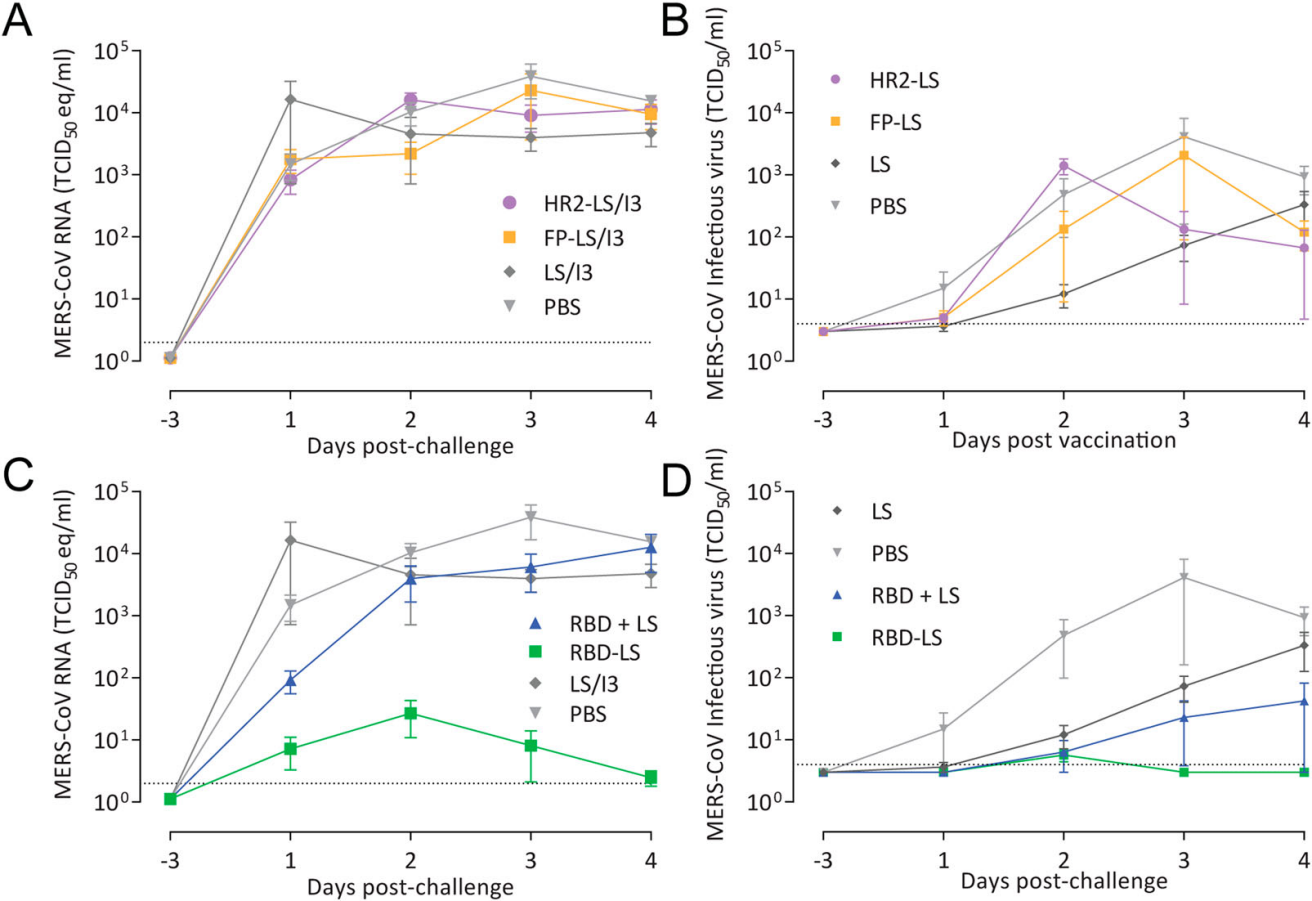
Protective capacity of MERS-CoV MPSP vaccines against upper respiratory tract infection in rabbits. Six groups of vaccinated and control rabbits (*n* = 5/group) were tested for the presence of viral RNA (A, C) and infectious virus particles (B, D) in the upper respiratory tract (nasal swabs) at days −3 and 1–4 post intranasal viral challenge (days 46 and 50–53 post first vaccination) with 10^6^ TCID_50_ MERS-CoV EMC strain. Shown is the average ± s.e.m. of five animals per group. The dotted lines represent the limits of detection. HR2, hepad repeat 2; FP, fusion peptide; LS, lumazine synthase 60-meric particles; I3, I3-01 60-meric particles; RBD, receptor binding domain; RBD + LS, monomeric uncoupled RBD; RBD-LS, multimeric RBD coupled to LS through covalent SpyTag/SpyCatcher.

**Figure 6.**
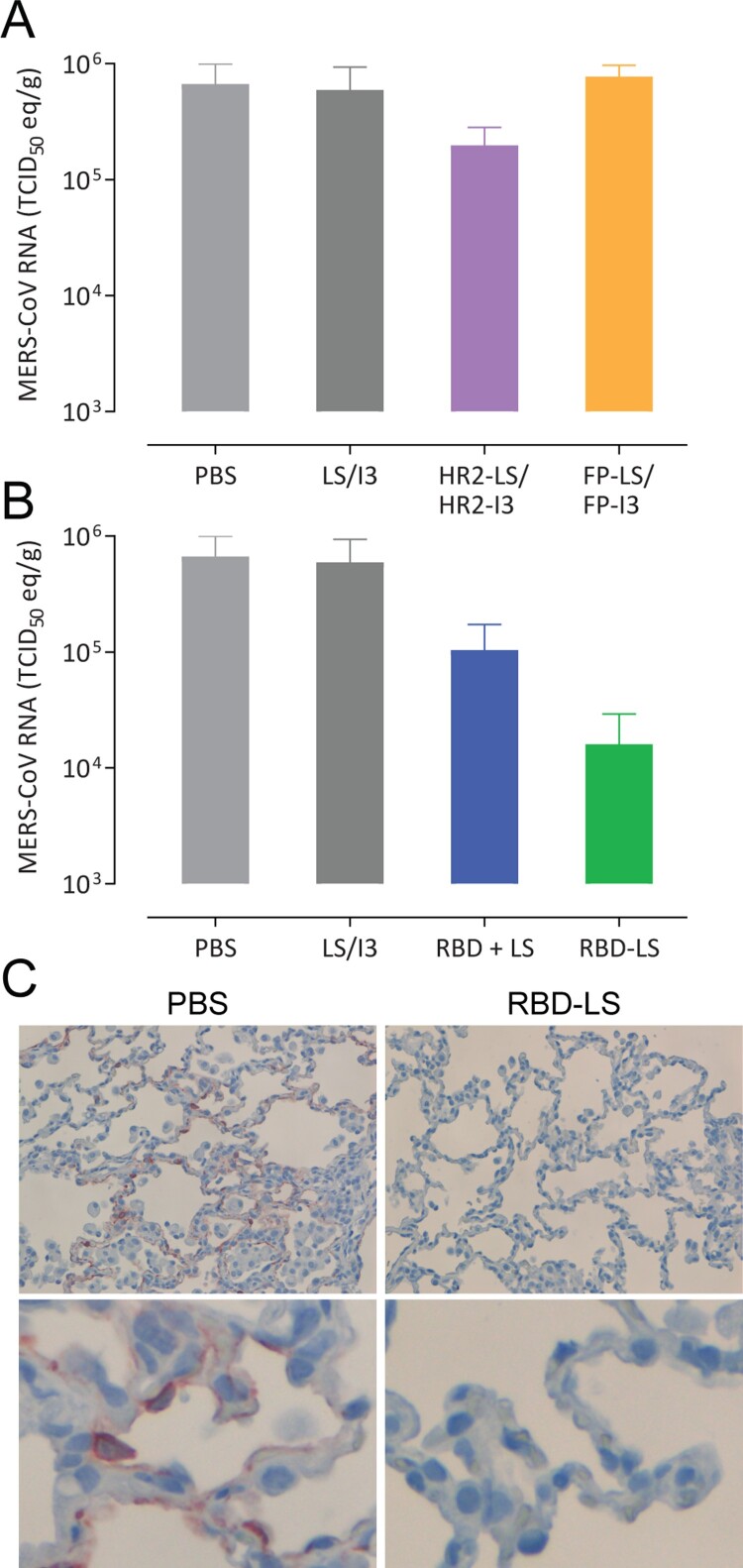
Protective capacity of MERS-CoV MPSP vaccines against lower respiratory tract infection in rabbits. Six groups of vaccinated and control rabbits (*n* = 5/group) were tested for the presence of viral RNA (A, B) in the lung tissue homogenates and viral nucleocapsid antigen in lung tissues (C) collected 4 days post intranasal viral challenge with 10^6^ TCID_50_ of MERS-CoV (EMC isolate). (A, B) Shown are the average and SEM equivalent virus titres/gram of tissue. C) Representative pictures of immunohistochemical detection of MERS-CoV nucleoprotein (shown in red) in the lungs of PBS (left) vs RBD-LS (right) immunized rabbits four days post-viral challenge; the upper and lower panels show a 200X and 1000X magnification, respectively. HR2, hepad repeat 2; FP, fusion peptide; LS, lumazine synthase 60-meric particles; I3, I3-01 60-meric particles; RBD, receptor binding domain; RBD + LS, monomeric uncoupled RBD; RBD-LS, multimeric RBD coupled to LS through covalent SpyTag/SpyCatcher.

To evaluate the protective efficacy of the immune responses induced by the different MERS-CoV spike MPSP vaccines, rabbits were challenged intranasally with 10^6^ TCID_50_ of MERS-CoV (strain HCoV-EMC/2012) and nasal swabs were collected up to 4 days post inoculation (pi) (Figure 2A). On day 4 pi, the animals were euthanized, and lung tissue samples were collected. Consistent with earlier reports [34,35], none of the rabbits in the control group developed any clinical signs of infection upon MERS-CoV inoculation, and titration of infectious virus from lung tissues and nasal swabs was variable. Thus, to evaluate protection, we tested for MERS-CoV RNA by qRT-PCR, for MERS-CoV infectious virus by virus titration, and for MERS-CoV antigen (N protein) in lung tissues by immunohistochemistry (IHC). Except for the RBD-LS vaccinated group, viral RNA was detected in all vaccinated groups from day 1 through day 4 post-challenge at levels similar to control groups (Figures 5 and 6). Viral RNA titres were significantly reduced in the nasal swabs of the RBD-LS vaccinated groups as early as day 1 post-challenge and were undetectable by day 4, in line with the absence of detectable infectious virus particles (Figure 5). Viral RNA was also reduced in the lungs of RBD-LS-vaccinated rabbits (Figure 6). Consistently, IHC revealed no viral antigen in the lungs of the RBD-LS vaccinated rabbits (Figure 6C), and antigen was also not detected in the RBD + LS vaccinated rabbits. Overall, in contrast to the monomeric form, the antigen-focused multimeric RBD-LS vaccine was able to block MERS-CoV replication significantly in the nose and lungs of the infected rabbits.

The efficacy of RBD-LS immunization in protecting against a MERS-CoV challenge, makes it a potential vaccine candidate. However, for production at industrial scale, unnecessary sequences (e.g. tags) need to be removed, preparations have to be further structurally and biochemically characterized.

## Discussion

Recombinant subunit proteins provide advantages regarding safety, costs, and speed of vaccine production, making them very attractive platforms for the development of vaccines for emerging viruses. Multivalent antigen display allows for virus-mimicking presentation of antigens and has been shown to induce antibodies of high avidity and magnitude [7,10,11,27,36]; with non-viral self-assembling MPSP providing advantages over other multimeric antigen presentation platforms [8,12]. Among the MERS-CoV vaccine candidates developed so far, the latter approach has been used to design two candidates, both are based on the receptor-binding domain [27,37], the main target for MERS-CoV protective antibodies [26]. One used self-assembling ferritin nanoparticles [27] and the second used canine parvovirus (CPV) VP2 structural protein forming virus like particles [37] as scaffolds. Both vaccine candidates were able to induce humoral and cellular immune responses in mice, nonetheless none has been tested for its protective capacity in a viral-challenge animal model. In our study, using an immune-focusing approach to target protective epitopes and domains along with multivalent presentation on self-assembling LS particles using a spontaneous covalent linker (SpyTag/SpyCatcher). We report for the first time the *in-vivo* protective capacity of a multimeric MERS-CoV RBD particle vaccine.

We used self-assembling LS and I3 particles to generate chimeric multimeric protein scaffold particle displaying critical domains in the MERS-CoV spike protein and evaluated their immunogenicity and protective efficacy in rabbits. Multimeric FP and HR2 vaccinations induced high levels of anti-S2 antibodies, nonetheless, with low to undetectable virus neutralizing capacities and couldn’t protect rabbits against virus challenge. Meanwhile, multimeric RBD-LS vaccination was highly immunogenic and induced robust antibody responses of high magnitude, avidity and neutralizing capacity. Following a live virus challenge, it protected upper and lower respiratory tract of rabbits as detected by decrease in viral RNA titres, with an associated lack of MERS-CoV antigen (Figures 5 and 6). Despite producing strong antibody responses, the monomeric RBD failed to protect rabbits against MERS-CoV following an intranasal challenge. The presence of LS did not seem to influence the outcome, as it was included in the formulation of the monomeric form (RBD + LS), indicating that the coupling and the multimeric presentation are responsible for the enhanced response seen with the multimeric RBD-LS vaccine. The “plug-and-display” SpyTag/SpyCatcher system [8] used to generate these multimeric RBD-LS particles allows for rapid and robust production of vaccines in a cost-effective manner. This enables the development of vaccines in a timely manner, which is crucial to prevent global public health consequences of evolving, emerging and re-emerging viruses.

The efficacy of RBD-LS immunization in protecting against a MERS-CoV challenge, makes it a potential vaccine candidate for further development. Nonetheless, in case of production at an industrial scale, unnecessary sequences (e.g. tags) need to be removed, preparations have to be further structurally and biochemically characterized.

When using scaffolds as antigen carriers, anti-scaffold antibody responses need to be considered to avoid their potential to compromise the targeted antigen-induced responses or to induce potential auto-antibodies against human antigens. Antibody responses were induced against the LS protein scaffold used in this study. However, antigen-specific responses were boosted following the second immunization and were not adversely affected by the presence of these anti-scaffold antibodies (Figure 4), similar to other reports [38]. Since the sequence of the LS protein does not show any similarity to any human sequences, it is unlikely that they will induce unwanted auto- (antihuman) antibodies. An LS-based vaccine for HIV, in a current phase 1 clinical trial (NCT03547245), can provide further evidence for the safety of this platform. Nonetheless, we developed a heterologous scaffold prime-boost using LS and I3 which can help in reducing anti-scaffold responses.

A challenge facing MERS-CoV vaccine development is the limited number of appropriate animal models for testing protection against clinical virus isolates. Rabbits provide some advantages as an animal model for MERS-CoV. By having the MERS-CoV receptor DPP4 expressed in both the upper and lower respiratory tract epithelium [24], the rabbits can be naturally infected. This allows the evaluation of both upper and lower respiratory tract MERS-CoV infection and in turn protection using natural field virus isolates rather than adapted strains. However, the animals are not able to develop severe infection such as that seen in severe human cases [34]. Nonetheless, severe infection, thus far, has not been established consistently in any of the other animal models without genetic modification and/or virus adaptation, except for marmosets [39]. In addition to the aforementioned, rabbits are readily available and easier to handle compared to other species that can be naturally infected such as non-human primates.

Following the addition of MERS-CoV as a priority pathogen in the WHO R&D Blueprint for action to prevent epidemics, a target product profile was developed which called for three types of MERS-CoV vaccines [40]. These include one for camels to prevent virus shedding and transmission, and two for humans: a two-dose vaccine for long-term protection of those at continuous high risk such as camel handlers and health-care workers, and a single-dose vaccine for rapid onset of immune responses to protect those at acute risk in outbreak settings. The RBD-LS can be used to develop the two-dose vaccine required to protect the high-risk populations, and can be further optimized using the heterologous scaffold prime/boost scheme developed in this study. Nonetheless, evaluating the longevity of the induced immune responses is warranted. Following the prime, RBD-LS vaccination induced antibody responses of high avidity and MERS-CoV neutralizing capacity. Owing to the robust immune responses induced after one dose, the RBD-LS can be a candidate for developing a rapid single-dose vaccine for MERS-CoV, which is required for reactive use in outbreak situations [40]. Additionally, this vaccine candidate was able to block MERS-CoV replication in the upper respiratory tract of infected rabbit, thus it could potentially be of use as a dromedary vaccine to block MERS-CoV transmission. However, both approaches need to be further validated.

## Contributions

B.L.H. and B,j.B, designed the study. N.M.A.O., I.W., B.v.D, G.v.A, L.d.W., K.j.S., D.S., J.v.d.B, B.M. performed the studies. N.M.A.O. designed experiments and analyzed the data. A.A. and M.B. provided the LS-SC. N.M.A.O, B.j.B and B.L.H. wrote the manuscript with comments from all co-authors.

## Supplementary Material

Supplemental Material

## Data Availability

All data are available within the article and its supplementary information or available from the authors on request.
