# Supplementary material for: Particulate multivalent presentation of the receptor binding domain induces protective immune responses against MERS-CoV
Source: Emerg Microbes Infect. 2020 May 29;9(1):1080–91. doi: 10.1080/22221751.2020.1760735 (PMC7448924; doi:10.1080/22221751.2020.1760735)

Supplementary Materials

**Table S1.** Immunogens used in this sudy

| Protein | Number of amino acids | Size (KDa) |
| --- | --- | --- |
| LS | 194 | 20.47 |
| I3 | 226 | 24.10 |
| FP-LS | 210 | 22.27 |
| FP-I3 | 261 | 27.71 |
| HR2-LS | 257 | 27.46* |
| HR2-I3 | 319 | 34.07 |
| RBD-ST | 286 | 30.34* |
| LS-SC | 270 | 28.80 |
| * These proteins appear larger in SDS-PAGE analysis due to N-glycosylation of HR2/RBD domain.  FP, MERS-CoV fusion peptide; HR2, MERS-CoV heptad repeat 2; I3, I3-01; LS, Lumazine synthase; MERS-CoV, Middle East respiratory syndrome coronavirus, RBD, MERS-CoV receptor binding domain; SC, SpyCatcher, ST, SpyTag. | | |

**Table S2.** Vaccine-induced antibody titers and fold changes following prime (4 weeks post-prime) and booster (3 weeks following booster) vaccinations

|  | S1/S2 antibody titers | | | | Neutralizing antibody (PRNT_90_) titers | | | |
| --- | --- | --- | --- | --- | --- | --- | --- | --- |
|  | **Geometric mean titer**  **GMT (95% CI)** | | **Fold increase in titer**  **(95% CI)** | | **Geometric mean titer GMT (95% CI)** | | **Fold increase in titer**  **(95% CI)** | |
|  | **Prime** | **Boost** | **Prime** | **Boost** | **Prime** | **Boost** | **Prime** | **Boost** |
| HR2-LS/I3 | 6.4 x10^3^  (6.4-6.4 x10^3^) | 2.6 x10^4^  (2.6 x10^4^-2.6 x10^4^) | 128  (128-128) | 512  (512-512) | N/A | 22.97  (11.2-47.2) | N/A | 2.297  (1.1-4.72) |
| FP-LS/I3 | 200  (59.21-675.5) | 1.5 x10^4^  (5.7 x10^3^-3.8 x10^4^) | 4  (1.2-13.5) | 294.1  (114.5-754.9) | N/A | N/A | N/A | N/A |
| LS/I3 | neg | neg | neg | neg | N/A | N/A | N/A | N/A |
| PBS | neg | neg | neg | neg | N/A | N/A | N/A | N/A |
| RBD+LS | 4.9 x10^3^  (2.2 x10^3^-1 x10^4^) | 5.9 x10^4^  (2.3 x10^4^-1.5 x10^5^) | 97.01  (44.9-209.5) | 1176  (458.2-3020) | 30.3  (11.4-80.9) | 422.2  (195.5-911.8) | 3.031  (1.1-8.1) | 42.22  (19.6-91.2) |
| RBD-LS | 1.9 x10^4^  (9 x10^3^-4.2 x10^4^) | 4.1 x10^5^  (1.2 x10^5^-1.4 x10^6^) | 388  (179.7-837.9) | 8192  (2.4 x10^3^-2.8 x10^4^) | 320  (174.1-588.1) | 4208  (2883-6141) | 32  (17.4-58.8) | 420.8  (288.3-614.1) |
| *Antibody titers and fold increase relative to baseline (Day 0) are expressed in GMT (95% CI) for n=5 rabbits/group. CI, confidence interval; GMT, geometric mean titer FP, MERS-CoV fusion peptide; HR2, MERS-CoV heptad repeat 2; I3, I3-01; LS, Lumazine synthase; MERS-CoV, Middle East respiratory syndrome coronavirus, N/A, not applicable; neg, negative; PRNT_90_, 90% reduction in plaque reduction neutralization test using MERS-CoV EMC strain; RBD, MERS-CoV receptor binding domain; S1, MERS-CoV Spike protein S1 subunit; S2, MERS-CoV spike protein S2 subunit; SC, SpyCatcher, ST, SpyTag.* | | | | | | | | |

**Fig. S1.** Domains of the MERS-CoV spike protein that are presented on multimeric protein scaffold particles. Domains are color-coded: receptor binding domain (RBD, green), fusion peptide (FP, orange), Heptad repeat 2 (HR2, lilac).


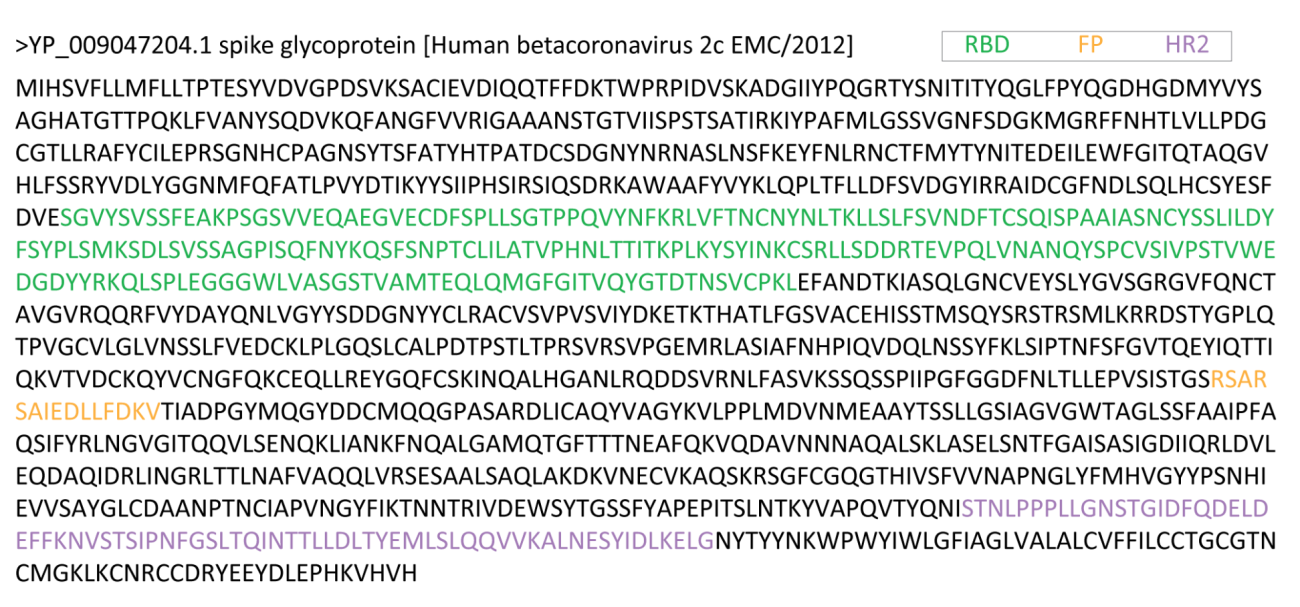


**Fig. S2.** Amino acid sequences of protein constructs used in this study for immunization of rabbits. Different domains are color-coded: signal sequence (grey), lumazine synthase (LS, orange), I3-01 (I3, pea green), Streptag (green), SpyTag (ST, light blue), SpyCatcher (SC, dark blue), MERS-FP (FP, lilac), MERS-HR2 (HR2, red), MERS-CoV RBD (RBD, purple).


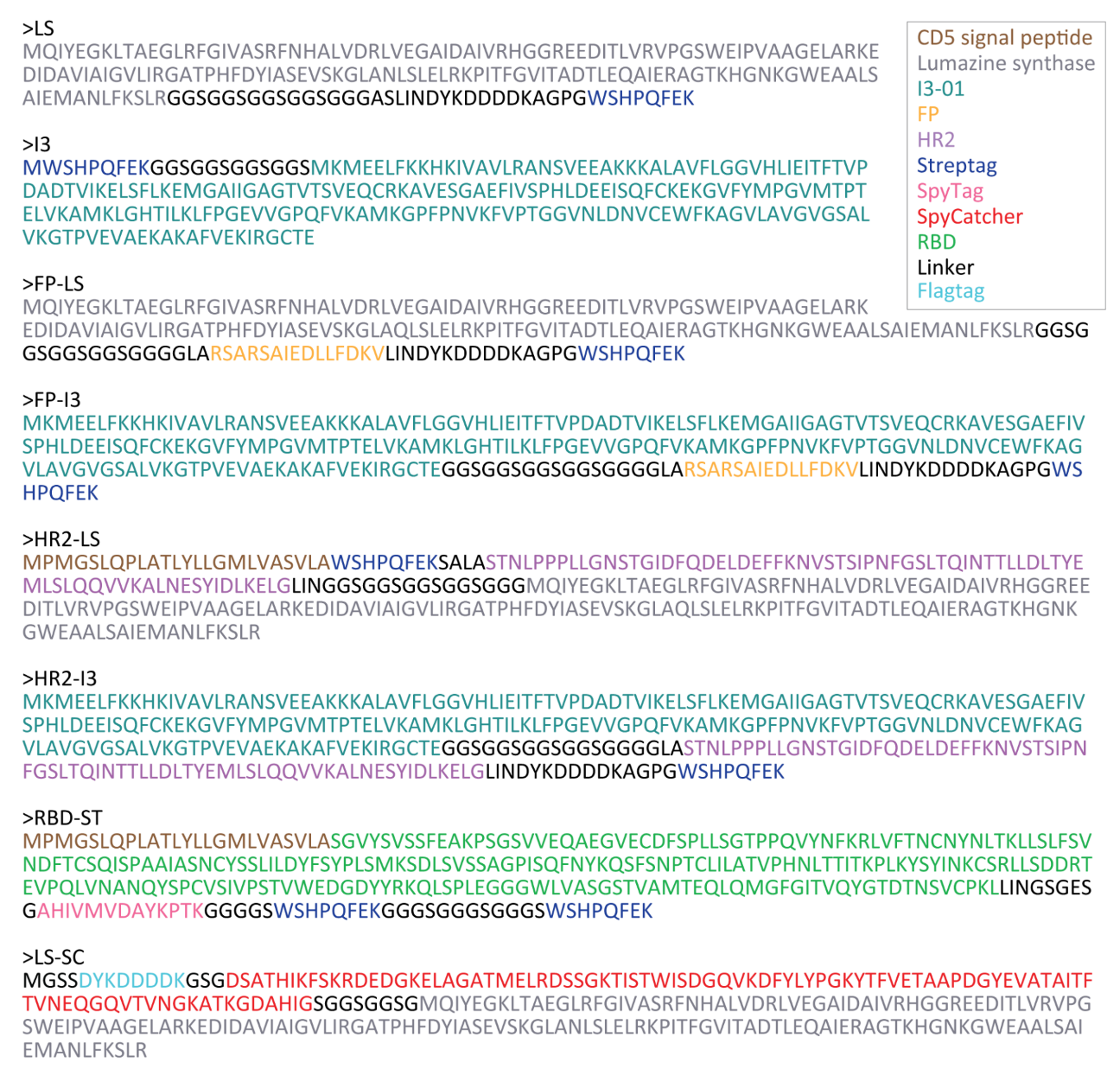


**Fig S3.** Binding of MERS-CoV RBD-LS particles by RBD-specific human monoclonal antibodies.

**
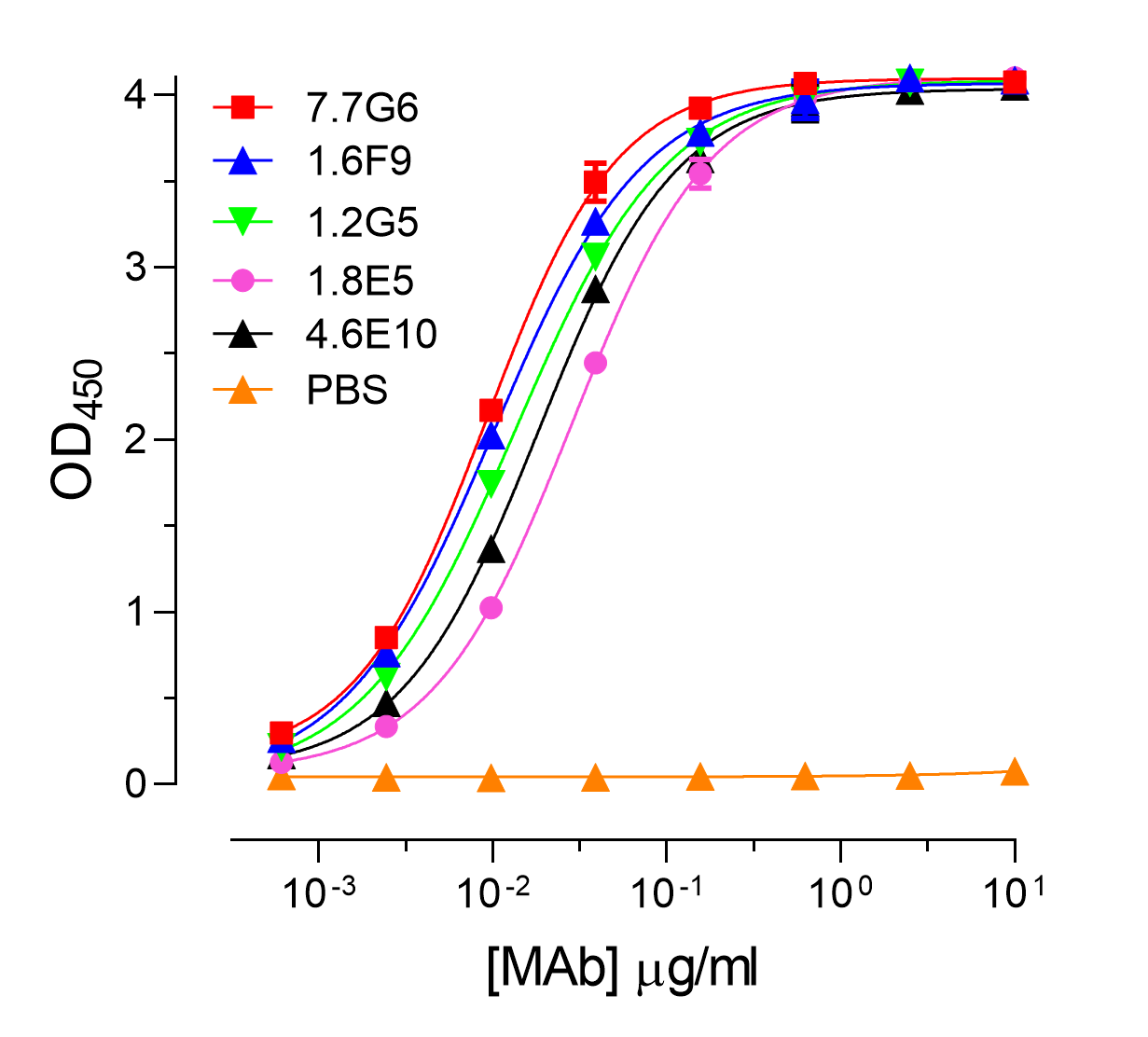
**

**Fig. S4.** Vaccine-induced MERS-CoV neutralizing antibodies in sera of vaccinated rabbits against the clade B Qatar15 strain (GenBank accession no. MK280984.2) using plaque reduction neutralization assay (PRNT). The dotted line represents the lower limit of detection.


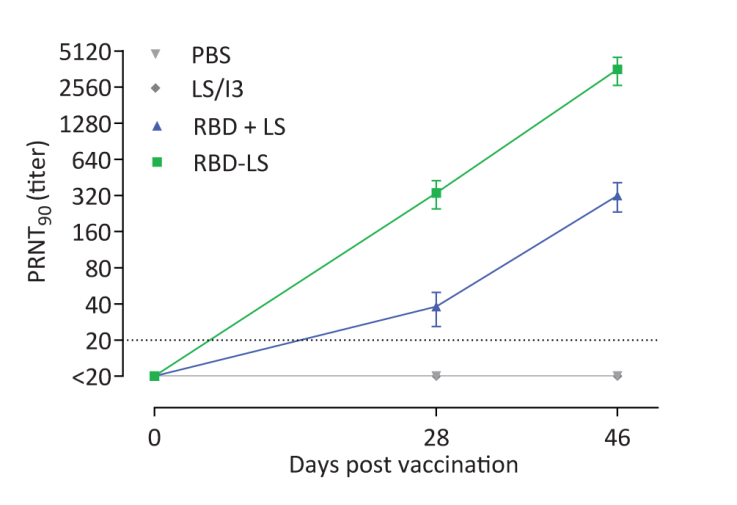

Supplement: Supplemental Material [file TEMI_A_1760735_SM7168.docx]
